# Enzymolytic Lignin-Derived N-S Codoped Porous Carbon Nanocomposites as Electrocatalysts for Oxygen Reduction Reactions

**DOI:** 10.3390/ma16247614

**Published:** 2023-12-12

**Authors:** Zheng Li, Xia Qu, Yuwei Feng, Lili Dong, Yantao Yang, Tingzhou Lei, Suxia Ren

**Affiliations:** 1 Institute of Urban & Rural Mining, Changzhou University, Changzhou 213164, China; lizheng04192023@163.com (Z.L.); qx2621903540@163.com (X.Q.); f3547932934@163.com (Y.F.); dongli2050@cczu.edu.cn (L.D.); yyt@cczu.edu.cn (Y.Y.); 2 Changzhou Key Laboratory of Biomass Green, Safe & High Value Utilization Technology, Changzhou 213164, China

**Keywords:** oxygen reduction reaction, nitrogen and sulfur double doping, lignin, carbon materials

## Abstract

Currently, the development of nonmetallic oxygen reduction reaction (ORR) catalysts based on heteroatomic-doped carbon materials is receiving increaseing attention in the field of fuel cells. Here, we used enzymolytic lignin (EL), melamine, and thiourea as carbon, nitrogen, and sulfur sources and NH_4_Cl as an activator to prepare N- and S-codoped lignin-based polyporous carbon (ELC) by one-step pyrolysis. The prepared lignin-derived biocarbon material (ELC-1-900) possessed a high specific surface area (844 m^2^ g^−1^), abundant mesoporous structure, and a large pore volume (0.587 cm^3^ g^−1^). The XPS results showed that ELC-1-900 was successfully doped with N and S. ELC-1-900 exhibited extremely high activity and stability in alkaline media for the ORR, with a half-wave potential (E_1/2_ = 0.88 V) and starting potential (E_onset_ = 0.98 V) superior to those of Pt/C catalysts and most non-noble-metal catalysts reported in recent studies. In addition, ELC-1-900 showed better ORR stability and methanol tolerance in alkaline media than commercial Pt/C catalysts.

## 1. Introduction

In the past few decades, the extraction and consumption of fossil fuels such as coal, oil, and natural gas have caused problems such as energy depletion and environmental pollution. In order to achieve carbon peaking and carbon neutrality goals, various countries have made tremendous efforts to develop advanced energy conversion and storage equipment [1]. Among these, fuel cells have attracted much attention due to their high energy density and high specific capacity, and their performance is superior to commercial Li-ion batteries [2]. However, the slow cathodic kinetics of oxygen reduction reaction (ORR) greatly limits the overall reaction rate of the battery, resulting in insufficient energy conversion efficiency of zinc air batteries [3,4], which also affects the further improvement of battery performance. Therefore, the way to improve battery efficiency is to enhance the oxygen reduction reaction of the cathode, which involves adding an oxygen reduction reaction catalyst to the cathode material. As a result, finding efficient and durable ORR electrocatalysts has become crucial. As is well known, platinum metals and platinum-based materials are still considered the most effective ORR catalysts [5]. However, due to low reserves, high costs, and poor cycle stability, Pt catalysts are difficult to meet the needs of large-scale applications [6,7]. In order to replace Pt catalysts, the development of nonprecious metal electrocatalysts has become a research hotspot.

Recently, the use of porous carbon materials has been recognized as an important direction in the field of new energy materials owing to their high specific surface area, adjustable pore structure and surface chemical structure, good conductivity, abundant raw material sources, and low cost. They have excellent potential to replace expensive Pt/C as ORR electrocatalysts at the cathode of fuel cells. The pore structure and surface chemical structure of carbon materials are the main structural factors that affect their performance as ORR electrocatalytic materials. Since the discovery of nitrogen-doped carbon nanotubes and graphene with high ORR electrocatalytic activity [8,9], the preparation of nitrogen-doped porous carbon has become the main research direction for the preparation of metal-free carbon material catalysts with high performance for the electrocatalytic ORR [10,11].

There have been many reports about lignin-based porous carbon materials as active electrode materials for energy devices such as supercapacitors [12], lithium-ion batteries, and sodium-ion batteries. There have been many reports on the use of carbon materials prepared from lignin as ORR electrocatalysts for fuel cell cathodes. In these studies, industrial lignins, such as alkali lignin [13] and lignosulfonate [14], were typically used as raw materials. First, heteroatoms such as sulfur and nitrogen were used in the raw materials [15], and then nitrogen compounds were added to pyrolyze the lignin to obtain carbon materials doped with different heteroatoms; alternatively, nitrogen-containing lignin was prepared by nitrification. Then, these lignin materials were carbonized or activated to prepare lignin-based porous carbon materials. For example, Zhang et al. synthesized N- and S-codoped carbon nanosheets by using alkaline lignin (AL) as the carbon source by a simple one-step pyrolysis method. Sample N-S-C 900 exhibited excellent electrocatalytic activity for ORR, which is better than the commercial Pt/C catalyst with regard to the half-wave potential and limiting current density in alkaline medium. At the same time, the obtained catalyst also showed better ORR stability and excellent methanol tolerance in alkaline and acidic media than commercial Pt/C catalysts [16]. Yan et al., using magnesium lignin sulfonate as a sulfur source and carbon precursor, melamine as a nitrogen source, MgO as a hard template, and ZnCl_2_ as an activator, synthesized nitrogen–sulfur-codoped three-dimensional magnesium lignin sulfonate (MLS-derived) floral-fractionated porous carbon (NSLPC) materials by a simple, green method. When the ratio of MgO to ZnCl_2_ was 10:0.5, NSLPC-1005 had the best ORR activity and a high specific surface area (1752.54 m^2^ g^−1^). In alkaline media, the initial potential of NSLPC-1005 was 0.97 V, and the half-potential was 0.86 V [17]. However, there have been few reports on the preparation of electrocatalytic materials for fuel cells by enzymatic hydrolysis of lignin. The chemical structure and physical state of lignin during enzymatic hydrolysis are obviously different from those of other industrial lignins due to the small amount of nitrogen atoms, high purity, and low impurity content. This material may be used to prepare ORR electrocatalytic carbon materials with excellent properties. However, the pore structure of lignin-based carbon modified by nitrogen-containing compounds is underdeveloped, and the specific surface area is low. A high specific surface area and developed pore structure are the basic prerequisites for the preparation of high-performance ORR electrocatalysts [18]. Therefore, an effective method is to use activators for activation. For example, Ma et al., using alkaline lignin as a raw material, MgO as a hard template, and KOH/NaOH as an activator, synthesized hierarchical porous nitrogen-doped floral carbon nanosheets and then deposited n-CQDs in situ on the carbon nanosheets by a one-step hydrothermal method to prepare n-HPFN@CQDs. Due to their abundant active sites, high specific surface area and developed pore structure, n-HPFN@CQDs showed better ORR catalytic activity and stability in a 0.1 mol KOH medium than commercial Pt/C catalysts [19]. Huang et al. prepared N-doped carbon nanosheets (LNZ@GC) by a two-step process of ball milling carbonization using alkali lignin as the carbon source and NH_4_Cl and ZnCl_2_ as activators. LNZ@GC showed excellent ORR performance, with a half-wave potential of up to 0.851 V, and excellent stability and methanol tolerance, both of which were superior to those of commercial Pt/C [20].

To prepare lignin-based activated carbon with a developed pore structure and high nitrogen content, ammonium chloride was used as an activator, melamine and thiourea were used as nitrogen and sulfur sources, and N- and S-codoped enzymolytic lignin (EL)-based porous carbon materials were prepared by a one-step pyrolysis method. The relationship between the pore structure, defect degree, form of nitrogen and sulfur doping, and ORR electrocatalytic performance was investigated by various characterization methods and electrochemical tests.

## 2. Experimental Section

### 2.1. Experimental Materials

EL is provided by the sub project of The National Key Research and Development Program of China (Structure Activity Relationship and Molecular Reconstruction Mechanism of Lignocellulose Component Transformation (2021YFC2101604)); melamine (C_3_H_6_N_6_, 99%), thiourea (CH_4_N_6_S, AR, 99%), ammonium chloride (NH_4_Cl, 99.5%) and ethanol (analytical purity, 99.7%) were purchased from McLean Chemical Reagent Co., Ltd., Shanghai, China; and Nafion (D520, 5%) was obtained from Shanghai Hesen Electric Co., Ltd., Shanghai, China.

### 2.2. Experimental Procedure

First, an amount of melamine (1~4 g) and thiourea (1~4 g) was dispersed into 100 mL of DMF and stirred at 100 °C for 30 min until they were completely dissolved. Afterwards, 1 g of EL and 1 g of ammonium chloride were added into the mixed solution with 30 min stirring. Then, the mixture was heated to 155 °C in order to evaporate most of the DMF solution. After that, the mixture was dried in a blower dryer for 12 h and powder solids were obtained. Finally, the obtained solids were heated to 900 °C at a heating rate of 5 °C/min and maintained for 2 h carbonization under nitrogen atmosphere in a tube furnace. The sample was further washed with hydrochloric acid and deionized water to neutral. Samples with mass ratios of lignin, melamine, thiourea, and ammonium chloride of 1:1:1:1, 1:2:2:1, and 1:4:4:1 were labelled ELC-1-900, ELC-2-900, and ELC-4-900, respectively. For comparison, samples prepared under the same conditions without the addition of melamine, thiourea, and ammonium chloride were labelled ELC-900. In addition, for ELC-1-900, we explored the effects of different carbonization temperatures (800 and 1000 °C), and the resulting samples were denoted as ELC-1-800 and ELC-1-1000, respectively.

### 2.3. Catalyst Structure Characterization

The surface element distribution and valence states of the samples were determined by X-ray photoelectron spectrometer (XPS, Thermo Scientific K-Alpha, Waltham, MA, USA). Raman spectroscopy was used to characterize the material defects from 800 to 2000 cm^−1^ on LabRAM Aramis (Renishaw, inVia Raman microscope, Wotton-under-Edge, UK) with an excitation wavelength of 532 nm. The morphologies of the carbon materials were characterized by high-resolution transmission electron microscopy (HRTEM, FEI Tecnai F20, Hillsboro, OR, USA). The specific surface area and pore size distribution of the samples were obtained by the Brunauer–Emmett–Teller (BET) way and the Barrett–Joyner–Halenda (BJH) theory (BET, MAC, TriStar II 3flex, New York, NY, USA).

### 2.4. Electrochemical Measurements

Electrochemical tests were carried out in a three-electrode cell at room temperature by means of the electrochemical workstation (CHI 760E, Shanghai, China). In this experiment, a rotating disk electrode (RDE) served as the working electrode, the platinum sheet electrode was used as the counter electrode, and a saturated calomel electrode (SCE) was taken as the reference electrode. Among them, the diameter of the RDE glassy carbon (GC) is 4 mm. The test was conducted in an alkaline environment with an electrolyte solution of 0.1 M KOH.

The electrocatalyst ink was prepared by taking 2 mg catalysts and dispersing them in a mixture solution, which contained 250 μL deionized water, 250 μL ethanol, and 50 μL Nafion (5 wt%); then, the ink was put in the ultrasonic cleaning machine for 40 min. Accordingly, 12 μL ink was dropped on the GC electrode and dried naturally to prepare the working electrode. As a comparison, the commercial Pt/C (Johnson Matthey, 20 wt%) catalysts were tested by using the above method.

Linear sweep voltammetry (LSV) and cyclic voltammetry (CV) curves of catalyst samples were obtained in 0.1 M KOH solution saturated with N_2_ or O_2_. The potential range of CV was 0–1.2 V and the scanning rate was 50 mV s^−1^. Similarly, the testing potential range of LSV was 0–1.2 V but the scanning rate was 5 mV s^−1^, all of which were completed in O_2_ atmosphere. The ORR polarization curve rotation speeds were set at 400, 900, 1600, and 2500 rpm. In the absence of other explanations, the LSV of 1600 rpm was selected for comparison in this paper.

The chronoamperometric method (I-t) was tested in 0.1 M KOH electrolyte saturated with O_2_. The speed of RDE is 1600 rpm and the durability of the catalyst samples were evaluated after continuous operation for 10000 s. After polarizing the working electrode for 300 s, 3 M methanol was added to the electrolyte and the remaining 700 s of current changes were monitored. The smaller the current change, the better the methanol resistance of the catalyst.

## 3. Results and Discussion

### 3.1. Characterization of Different Catalysts

**Figure 1:**
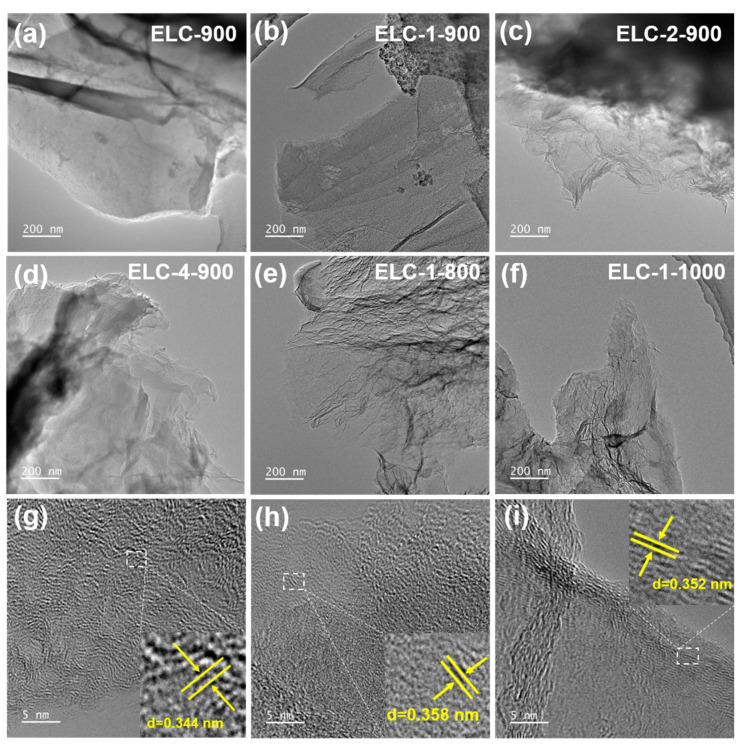
TEM images of samples with different catalysts: (**a**) ELC-900, (**b**) ELC-1-900, (**c**) ELC-2-900, (**d**) ELC-4-900, (**e**) ELC-1-800, and (**f**) ELC-1-1000. High-resolution TEM images: (**g**) ELC-1-900, (**h**) ELC-1-800, and (**i**) ELC-1-1000.

TEM was used to analyze and characterize the microstructure and crystal parameters of the catalyst samples. In addition to ELC-900, other samples showed a graphene-like transparent structure. Next, we discuss the influence of temperature on the sample morphology. In the ELC-1-800, ELC-1-900, and ELC-1-1000 samples, many folds were observed on the surface of the carbon nanosheets (Figure 1b,e,f), which further increased the specific surface area and changed the defect degree of graphitic carbon [21]. This porous and folded structure facilitated the occurrence of a high proportion of exposed edge sites, a high density of active sites, and a large surface-to-mass ratio to enhance catalytic activity.

From the high-resolution TEM images, it can be seen that the catalyst samples ELC-1-800, ELC-1-900, and ELC-1-1000 all possessed clear lattice fringes with lattice spacings of approximately 0.34~0.35 nm, corresponding to the (002) crystal face of graphitic carbon [22], which improved their electronic conductivity. This structure resulted in more active sites for electrocatalytic reactions [23].

**Figure 2:**
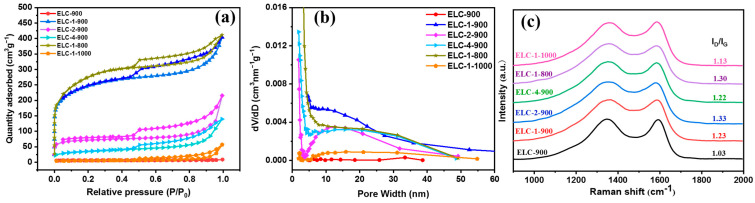
(**a**) N_2_ adsorption–desorption isotherms; (**b**) aperture distribution curves for different catalysts; (**c**) Raman spectra of the catalyst at different temperatures and ratios.

ORR catalytic reactions occurred on the surface of catalysts, and the specific surface area of the catalyst is a key factor. The larger the specific surface area, the more favorable it is for the exposure of active sites, thereby improving the ORR catalytic performance [24]. In order to analyze the effect of N and S doping on the specific surface area and pore size of the catalyst, we tested and characterized the N_2_ adsorption and desorption isotherms of the catalyst. Figure 2a shows the N_2_ adsorption and desorption curves of different catalysts. All the samples except for ELC-900 showed type IV isotherm characteristics in the middle- and high-pressure regions (P/P_0_ = 0.45–1), and there were obvious retention rings, indicating rich mesoporous structures [25]. The presence of these mesopores enhances the mass transfer effect of the ORR, facilitating the progress of the ORR.

The specific surface areas of different catalysts were characterized by BET. Among them, the specific surface areas of ELC-900, ELC-1-900, ELC-2-900, ELC-4-900, ELC-1-800, and ELC-1-1000 were 18, 844, 262, 123, 962, and 18 m^2^ g^−1^, respectively (Table 1). Compared with that of the EL carbon material, the specific surface areas of the samples doped with N and S were improved. NH_3_ and other gases produced during the decomposition of melamine and thiourea in the carbonization process etched the surface of carbon materials at high temperature and played a key role in the formation of pore channels as expansion agents. The pore size distributions of different catalysts were determined by means of the Barrett–Joyner–Halenda (BJH) equation. The average aperture size of ELC-900 was 4.22 nm, while the average aperture sizes of ELC-1-900, ELC-2-900, ELC-4-900, ELC-1-800, and ELC-1-1000 were 4.36~11.56 nm. The NH_3_ produced during the carbonization process not only increased the specific surface area but also further enlarged the average pore diameter of the samples.

Figure 2c shows that the spectra of all the samples have two characteristic peaks corresponding to the D band and the G band at 1350 cm^−1^ and 1590 cm^−1^, respectively [26]. The D band indicates disordered carbon or defects in sp^3^-hybridized carbon, which may be related to vacancy and heteroatom doping. The G band is caused by the in-plane tensile vibrations of sp^2^-hybridized carbon atoms (including C-C and N-C) in the graphite-carbon structure [27]. Generally, the ratio of the intensity of the D band to that of the G band (I_D_/I_G_) reflects the defect degree and graphitization degree of carbon materials. Compared with that of ELC-900 (1.03), the ratio of the D band to the G band of ELC-1-900 (1.23) was 0.2 higher, indicating that the defect concentration of ELC-1-900 was relatively high, and there were many defects or distorted structures. The presence of these structures is conducive to enhancing the catalytic performance of the sample. In general, having a high I_D_/I_G_ value endows carbon materials with excellent ORR properties [28].

**Figure 3:**
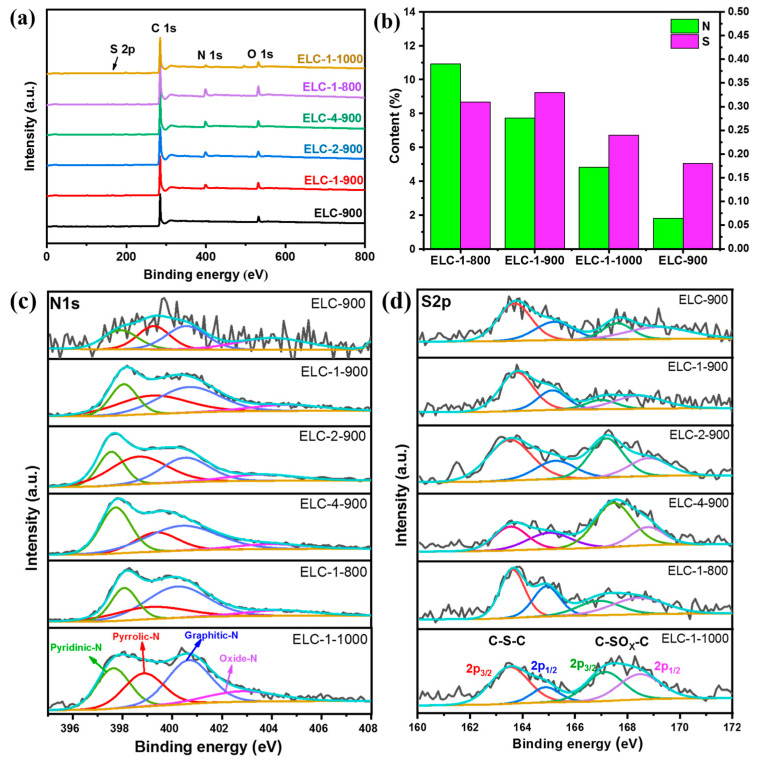
(**a**) Full-scan XPS spectra, (**b**) nitrogen and sulfur contents, (**c**) high-resolution N1s, and (**d**) S2p spectra of different catalysts.

Heteroatom doping may affect the electronic structure of carbon atoms and, thus, further affect the catalytic ORR activity of the material. Therefore, the contents of nitrogen and sulfur and their chemical valence states in different samples were characterized by XPS, and the results are shown in Figure 3. It can be seen from Figure 3a that the spectra of ELC-900, ELC-1-900, ELC-2-900, ELC-4-900, ELC-1-800, and ELC-1-1000 all have an obvious N1s (399.1 eV) characteristic peak, indicating the successful doping of N. The two characteristic peaks of C1s (284.8 eV) and O1s (532.1 eV) were observed for all samples. The characteristic peak of S2p (164.0 eV) was hardly observed in the spectra, which may be due to the low content of S compared with other elements, as seen in Table S1. In Figure 3b and Table S1, the proportion of elements was summarized and compared, and the S (0.33%) content of ELC-1-900 was the highest. The nitrogen contents of ELC-1-800, ELC-1-900, and ELC-1-1000 were 10.93%, 7.73%, and 4.83%, respectively. With increasing pyrolysis temperature, the total nitrogen content gradually decreased, indicating that a high carbonization temperature was not good for obtaining carbon materials with a high nitrogen content.

In general, there are four nitrogen-doped configurations on the carbon skeleton, namely, pyridinic nitrogen (pyridinic-N, 397.5–398.0 eV), pyrrolic nitrogen (pyrrolic-N, 398.8–399.3 eV), graphitic nitrogen (graphitic-N, 400.3–400.7 eV), and nitrogen oxide (oxidic-N, 403.3–404.0 eV) [29]. During the catalytic ORR, pyridinic-N and graphitic-N play important roles. The free electron pair of pyridinic-N can bind to the O_2_ molecule, thus facilitating its reduction on the adjacent C atom, while graphitic-N is thought to catalyze the reduction of O_2_ to H_2_O_2_. Therefore, the presence of graphitic-N increases the ORR limiting current density, while pyridinic-N is thought to help reduce the ORR overpotential. Figure 3c,d show high-resolution N1s and S2p spectra, which provide insight into the elemental distribution of nitrogen and sulfur, where ELC-1-900 had a high proportion of pyridinic-N (19.96%) and graphitic-N (38.05%), thus promoting the ORR four-electron pathway. In addition, the high-resolution S2p spectrum was deconvoluted into four peaks (Figure 3d), and the peaks at 163.5 eV and 165.0 eV were attributed to thiophene sulfur (-C-S-C-). The peak at 167.0–167.5 eV and 168.4–169.0 was attributed to sulfur oxide (-C-SOX-C-) [30,31], and thiophene sulfur was generally considered one of the active sites for the ORR in alkaline media [32]. The sulfur content of ELC-1-900 was the highest (0.33%), and thiophene sulfur accounted for 72.21%. With a further increase in temperature, due to the thermal instability of thiophene sulfur and sulfur oxide, the sulfur content of ELC-1-1000 was reduced to 0.24%, and the sulfur contents of ELC-1-800 and ELC-1-1000 were lower than that of ELC-1-900, indicating that an excessive carbonization temperature is not conducive to sulfur atom doping [21].

### 3.2. Electrocatalytic ORR Performance of Different Catalysts

**Figure 4:**
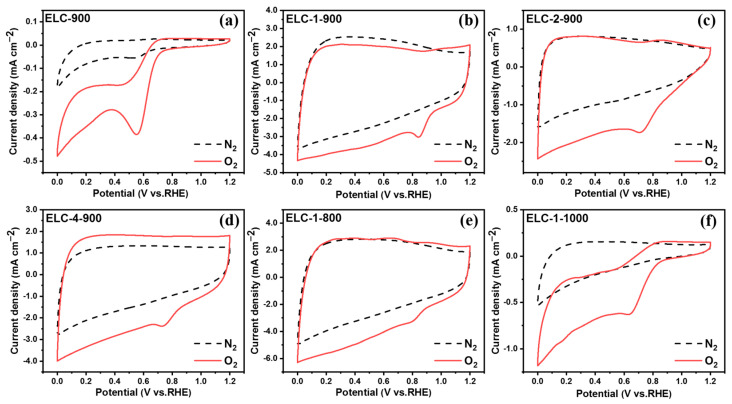
CV curves of different catalysts in an electrolyte saturated with O_2_ and N_2_; (**a**) ELC-900; (**b**) ELC-1-900; (**c**) ELC-2-900; (**d**) ELC-4-900; (**e**) ELC-1-800; (**f**) ELC-1-1000.

RDE and a three-electrode test system were used to test and analyze the electrocatalytic ORR performance of the sample, and the effect of nitrogen and sulfur doping on the electrocatalytic ORR performance of the catalyst was investigated. First, cyclic voltammetry of the sample in a 0.1 M KOH electrolyte saturated with O_2_ and N_2_ was performed. The results are shown in Figure 4, where the dashed line represents the results under N_2_ saturation and the solid line represents the results under O_2_ saturation. As seen from the figure, compared with the CV curve under nitrogen saturation, in the CV curves of ELC-900, ELC-1-900, ELC-2-900, ELC-4-900, ELC-1-800, and ELC-1-1000 under O_2_ saturation conditions, potentials of approximately 0.55, 0.84, 0.71, 0.74, 0.81, and 0.64 were obvious. The reduction peak indicated that the ORR occurred on the electrode, and the prepared catalyst samples exhibited electrocatalytic ORR activity. Among the samples, ELC-1-900 had a relatively high reduction peak potential, indicating that this sample had a higher ORR catalytic activity. There was no obvious redox peak in the CV curves obtained in N_2_-saturated electrolyte, and, in the O_2_-saturated electrolyte, there were significant redox peaks, indicating that these catalysts all have ORR activity.

**Figure 5:**
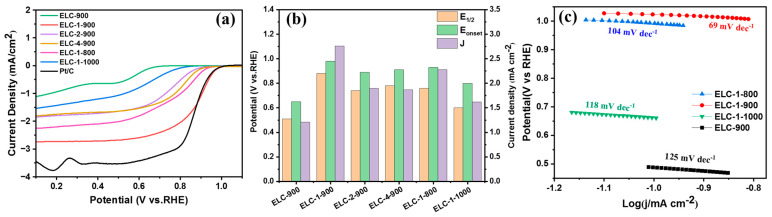
(**a**) LSV curves obtained for different samples at 1600 rpm; (**b**) ORR performance comparison bar chart; (**c**) Tafel slope.

To further investigate the ORR activity of the catalyst samples, the LSV curves of these materials in 0.1 M KOH oxygen-saturated electrolyte at room temperature were obtained, and the results are shown in Figure 5. The polarization curves of different catalysts at 1600 rpm are shown in Figure 5a. Figure 5b shows a bar chart of the half-wave potential, initial potential, and limit current density of different catalysts. The half-wave potential of different catalysts was very different, and the half-wave potential (E_1/2_) of the ELC-900 catalyst without any nitrogen source was only 0.51 V. After adding melamine and thiourea, the half-wave potentials of ELC-2-900 (E_1/2_ = 0.74 V) and ELC-4-900 (E_1/2_ = 0.78 V) were significantly increased. When the ratio of lignin to melamine to thiourea was 1:1:1 (ELC-1-900), the half-wave potential of ELC-1-900 was the highest, reaching 0.88 V, which is superior to commercial Pt/C catalysts (0.86 V).

To further investigate the effect of temperature on the ORR activity of the catalyst, we compared the ORR properties of ELC-1-900 with those of samples prepared at different temperatures but with the same component ratio: 800 °C (ELC-1-800) and 1000 °C (ELC-1-1000). The half-wave potential of ELC-1-900 was significantly higher than those of ELC-1-800 (E_1/2_ = 0.76 V) and ELC-1-1000 (E_1/2_ = 0.60 V). Considering the results of XPS analysis, although the ratio of graphitic-N to pyridinic-N in ELC-1-900 was lower than that in ELC-1-800, the proportion of thiophene sulfur in the sulfur content of ELC-1-900 (72.21%) was higher than that in ELC-1-800 (56.06%), indicating that thiophene sulfur resulted in higher ORR catalytic activity in alkaline media [21]. Figure 5c shows the Tafel slope of different catalysts in the kinetic stage of the ORR. The smaller the Tafel slope was, the faster the material progressed in the kinetic stage of the ORR. The Tafel slope of ELC-1-900 was 69 mV dec^−1^, which was lower than that of the other three samples, ELC-1-800 (104 mV dec^−1^), ELC-1-1000 (118 mV dec^−1^), and ELC-900 (125 mV dec^−1^), showing better kinetic characteristics. It can be seen from the above results that ELC-1-900 had the best ORR performance compared with other catalyst samples because it had a relatively high specific surface area and abundant mesopores. In addition, the doped nitrogen and sulfur provide active sites for the ORR performance of the material. It can be seen from the XPS results that ELC-1-900 had a relatively high nitrogen content (7.73%) and sulfur content (0.33%). Table S2 lists other reported catalysts and compares them with the ELC-1-900 catalyst prepared in this paper. It can be seen that the half-wave potential and initial potential of ELC-1-900 are high, making it a promising ORR catalyst.

**Figure 6:**
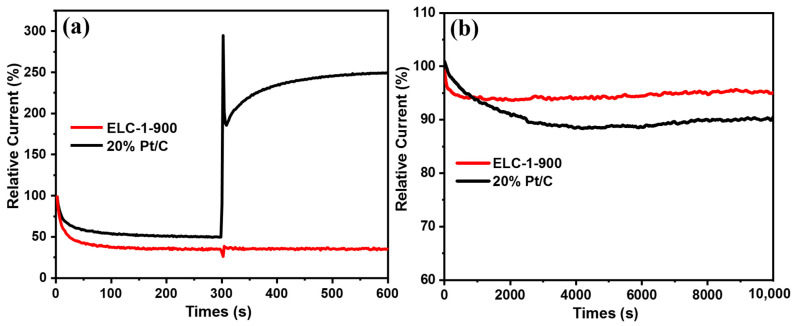
(**a**) Methanol tolerance and (**b**) stability test of ELC-1-900; test in an O_2_-saturated 0.1 M KOH solution (comparison to a commercial 20%Pt/C catalyst).

During the use of methanol fuel cells, the methanol fuel at the anode may have a toxic effect on the cathode catalyst, so the antimethanol crosslinking reaction ability of the catalyst is very important in practical applications. As shown in Figure 6a, 3 M methanol was added to the electrolyte during the chronocurrent test of the ELC-1-900 catalyst. When methanol was added, the current on the Pt/C electrode jumped significantly, while the current of ELC-1-900 changed very little. These results show that the ELC-1-900 catalyst had good antimethanol crosslinking ability and good selectivity for the ORR. In addition, stability is also an important index to evaluate the performance of ORR catalysts, and the stability of catalysts determines their service life in practical applications. Therefore, we performed stability tests of the ELC-1-900 sample with the best ORR catalytic activity and compared the results with those of a commercial Pt/C catalyst, and the results are shown in Figure 6b. After 10,000 s of continuous constant-voltage test, the current retention rate of ELC-1-900 was approximately 95.1%, which was higher than that of commercial Pt/C (89.9%), indicating that the ELC-1-900 catalyst had excellent stability. The above results show that the ELC-1-900 catalyst had better ORR catalytic activity, stability, and methanol tolerance than the commercial Pt/C catalyst in alkaline media.

## 4. Conclusions

In summary, we prepared N- and S-codoped porous carbon catalyst using enzymatic lignin as the raw material and NH_4_Cl as the activator by one-step pyrolysis method. The prepared porous carbon material has excellent electrocatalytic performance, indicating that enzymatic hydrolysis of lignin can also serve as an excellent carbon source material for preparing electrocatalysts. The BET results show that the catalyst has a high specific surface area of 844 m^−2^ g^−1^ and a large pore volume of 0.587 cm^3^ g^−1^, which provides as many active sites as possible for the catalyst. XPS analysis indicates that N and S doping was successfully carried out in the prepared sample. This N- and S-codoped carbon material shows good electrocatalytic activity for the ORR; its half-wave potential and initial potential are better than those of a Pt/C (20%) catalyst and it has better durability and methanol tolerance than a Pt/C catalyst, which makes the catalyst very promising for fuel cell applications.

## Figures and Tables

**Table 1:** The specific surface area, average pore diameter, and pore volume of different samples.

Samples	S_BET_ (m^2^ g^−1^)	Average Pore Diameter (nm)	Pore Volume (cm^−3^ g^−1^)
ELC-900	18	4.22	0.012
ELC-1-900	844	4.36	0.587
ELC-2-900	262	9.33	0.333
ELC-4-900	123	9.08	0.215
ELC-1-800	962	5.91	0.636
ELC-1-1000	18	11.56	0.088

## Data Availability

Data are contained within the article.

## Supplementary Materials

The following supporting information can be downloaded at: https://www.mdpi.com/article/10.3390/ma16247614/s1, Table S1: Contents of each element analyzed by XPS; Table S2: Comparison of the ORR properties of ELC-1-900 and other nonmetallic catalysts under alkaline conditions. References [33,34,35,36] are cited in the supplementary materials.
