# Supplementary material for: Enzymolytic Lignin-Derived N-S Codoped Porous Carbon Nanocomposites as Electrocatalysts for Oxygen Reduction Reactions
Source: Materials (Basel). 2023 Dec 12;16(24):7614. doi: 10.3390/ma16247614 (PMC10745107; doi:10.3390/ma16247614)
Supplement: Supplementary file 1 [file materials-16-07614-s001.zip › materials-2746565-supplementary.pdf]

Supporting information

Impact of different lignin sources on nitrogen-doped porous carbon toward the electrocatalytic oxygen reduction reaction

Zheng Li [1], Yuwei Feng [1], Xia Qu [1], Yantao Yang [1,2], Lili Dong[1,2], Tingzhou Lei [1,2]*, Suxia Ren [1,2]*

1 Institute of Urban & Rural Mining, Changzhou University, Changzhou 213164, China; s21020856156@smail.cczu.cn (Z.L.); s21020856056@smail.cczu.cn (Y.F.); s21020856027@smail.cczu.cn (X.Q.); yyt@cczu.edu.cn (Y.Y.); dongli2050@cczu.edu.cn (L.D.); leitingzhou@cczu.edu.cn (T.L.)

2 Changzhou Key Laboratory of Biomass Green, Safe & High Value Utilization Technology, Changzhou 213164, China

* Correspondence: rensuxia@cczu.edu.cn (S.R.); leitingzhou@cczu.edu.cn (T.L.).

1. Supplemental tables

Table S1 Contents of each element analysed by XPS

Sample	C (at%)	O (at%)	N (at%)	S (at%)
ELC-900	89.71	8.31	1.80	0.18
ELC-1-900	85.72	6.21	7.73	0.33
ELC-2-900	86.31	5.32	8.32	0.14
ELC-4-900	84.08	5.64	10.02	0.27
ELC-1-800	80.63	8.14	10.93	0.31
ELC-1-1000	88.18	6.75	4.83	0.24

Table S2 Comparison of the ORR properties of ELC-1-900 and other nonmetallic catalysts under alkaline conditions

Sample	$E_{1/2}$ (V)	E_{onset} (V)	Reference
N–S–C 900	0.83	0.97	[1]
N–S/C_700	0.75	0.80	[2]
Fe_2-N/CNTs-850 °C	0.846	0.972	[3]
Fe_5C_2/Mn, N, S-CNTs	0.85	1.04	[4]
AL-Ni/Co@GC	0.81	0.97	[5]
ELC-1-900	0.88	0.98	This work

2. References

1. Zhang, X. L.; Yu, D. L.; Zhang, Y. Q.; Guo, W. H.; Ma, X. X.; He, X. Q., Nitrogen- and sulfur-doped carbon nanoplatelets via thermal annealing of alkaline lignin with urea as efficient electrocatalysts for oxygen reduction reaction. *RSC ADVANCES* **2016,** *6* (106), 104183-104192.
2. Zhang, M.; Song, Y.; Tao, H.; Yan, C.; Masa, J.; Liu, Y.; Shi, X.; Liu, S.; Zhang, X.; Sun, Z., Lignosulfonate biomass derived N and S co-doped porous carbon for efficient oxygen reduction reaction. *Sustainable Energy & Fuels* **2018,** *2* (8), 1820-1827.
3. Li, C.; Wu, Y.; Fu, M.; Zhao, X.; Zhai, S.; Yan, Y.; Zhang, L.; Zhang, X., Preparation of Fe/N Double Doped Carbon Nanotubes from Lignin in Pennisetum as Oxygen Reduction Reaction Electrocatalysts for Zinc-Air Batteries. *ACS APPLIED ENERGY MATERIALS* **2022,** *5* (4), 4340-4350.
4. Wu, D.-H.; Huang, H.; Ul Haq, M.; Zhang, L.; Feng, J.-J.; Wang, A.-J., Lignin-derived iron carbide/Mn, N, S-codoped carbon nanotubes as a high-efficiency catalyst for synergistically enhanced oxygen reduction reaction and rechargeable zinc-air battery. *Journal of Colloid and Interface Science* **2023,** *647*, 1-11.
5. Dong, R.; Yang, Z.; Fu, Y.; Chen, Z.; Hu, Y.; Zhou, Y.; Qin, H., Aminated lignin chelated metal derived bifunctional electrocatalyst with high catalytic performance. *Applied Surface Science* **2022,** *580*, 152205.
